# Evaluation of the Combined Administration of *Chlorella fusca* and *Vibrio proteolyticus* in Diets for *Chelon labrosus*: Effects on Growth, Metabolism, and Digestive Functionality

**DOI:** 10.3390/ani13040589

**Published:** 2023-02-07

**Authors:** Jorge García-Márquez, Antonio Jesús Vizcaíno, André Barany, Alba Galafat, Gabriel Acién, Félix L. Figueroa, Francisco Javier Alarcón, Juan Miguel Mancera, Juan Antonio Martos-Sitcha, Salvador Arijo, Roberto Teófilo Abdala-Díaz

**Affiliations:** 1 Departamento de Microbiología, Facultad de Ciencias, Instituto Andaluz de Biotecnología y Desarrollo Azul (IBYDA), Universidad de Málaga, Ceimar-Universidad de Málaga, 29071 Málaga, Spain; 2 Departamento de Biología y Geología, Universidad de Almería, Ceimar-Universidad de Almería, 04120 Almería, Spain; 3 Department of Biology, Morrill Science Center, University of Massachusetts, Amherst, MA 01003, USA; 4 Departamento de Biología, Facultad de Ciencias del Mar y Ambientales, Instituto Universitario de Investigación Marina (INMAR), Universidad de Cádiz, Ceimar-Universidad de Cádiz, 11519 Cádiz, Spain; 5 Departamento de Ingeniería Química, Universidad de Almería, Ceimar-Universidad de Almería, 04120 Almería, Spain; 6 Departamento de Ecología y Geología, Facultad de Ciencias, Instituto Andaluz de Biotecnología y Desarrollo Azul (IBYDA), Universidad de Málaga, Ceimar-Universidad de Málaga, 29071 Málaga, Spain

**Keywords:** absorptive capacity, aquafeed, bacteria, enzymatic activity, fatty acids, fish quality, microalgae, Mugilidae, probiotic

## Abstract

**Simple Summary:**

The aquaculture industry is constantly looking for alternative ingredients to fishmeal and terrestrial-plant feedstuffs. Here, we have studied the effects of the combined administration of *Chlorella fusca* and ethanol-inactivated *Vibrio proteolyticus* DCF12.2 in the diet of the thick-lipped grey mullet (*Chelon labrosus*)*,* a promising candidate species for aquaculture diversification. The combined diet enhanced growth performance, n-3 polyunsaturated fatty acids, and fish lipid quality. Furthermore, the experimental diet increased several metabolic enzymes’ activity, as well as the intestinal absorption capacity. It can thus be concluded that the experimental diet composed of *C. fusca* and *V. proteolyticus* was suitable for feeding *C. labrosus.*

**Abstract:**

This study aimed to evaluate the combined effect of dietary *Chlorella fusca* and ethanol-inactivated *Vibrio proteolyticus* DCF12.2 (C + V diet) in *Chelon labrosus* juveniles, highlighting their nutritional, physiological, and morphological effects. The results showed that the combined dietary inclusion of *C. fusca* and *V. proteolyticus* significantly enhanced growth performance and feed utilization compared to the control group. The C + V diet increased the fish lipid quality index (FLQ), n-3 polyunsaturated fatty acids, and n-3/n-6 ratio, which might be beneficial in terms of human nutrition. The C + V diet considerably increased carbohydrate metabolic activity by statistically boosting plasma glucose. The dietary inclusion of *C. fusca* in conjunction with *V. proteolyticus* increased metabolic enzyme activity as well as intestinal absorption capacity compared to that found in the control group. In conclusion, the experimental diet was suitable for feeding *C. labrosus*, increasing their growth and the nutritional characteristics of the muscle and intestine, without causing tissue damage.

## 1. Introduction

Research into the use of microalgae as an alternative ingredient to fishmeal and terrestrial-plant ingredients has recently gained attention due to several features that make them attractive to the aquaculture industry [1]. Owing to their high nutritional value, *Chlorella* species have been used in the dietary formulations of several fish species, evidencing beneficial effects on growth, nutrient utilization, fatty acid content, and fish flesh quality, as well as improving their stress resistance and survival rates [2,3,4,5,6]. However, the nutritional effects of *Chlorella* species depend on the levels at which they are included in the aquafeeds. In this regard, Ahmad et al. [7] reviewed contradictory results on growth and feed efficiency in several fish species, the contradictions possibly being related to the inclusion level of the microalgae, since levels above 20% affected growth performance [8,9].

Probiotics are live microorganisms that confer a health benefit on the host when administered in adequate amounts [10,11]. Numerous studies have documented their application in human and farm animal health [12,13,14]. The beneficial features of probiotics include (i) improved nutrient supply to the host, (ii) an increase in aquafeed digestibility, (iii) the ability to colonize mucosal surfaces, acting as a barrier against pathogens, (iv) modulation of the gut microbiota, (v) the ability to inhibit bacterial growth through the production of bacteriocins and other substances, and (vi) immune response activation [15,16,17,18,19,20]. Despite these benefits, live probiotic cells pose certain safety and stability issues that have been highlighted before [21]. As an alternative to using live cells, it has been demonstrated that non-viable probiotic microorganisms can exert positive effects; these effects include improving growth performance [22,23], stimulating the host immune system [24], and modifying the intestinal microbiota [25]. In this context, non-viable microbial cells (or crude cell extracts) that confer positive effects on the host are referred to as “paraprobiotics” [26]. Paraprobiotics offer attractive advantages when it comes to industrial use. They make feed processing easier because they can be introduced before thermal procedures while still maintaining the level of activity necessary to provide the desired health benefits [27]. Paraprobiotics might also result in easier storage and administration, a longer shelf life, and provide supplementation for those with impaired immune systems [28,29].

In a previous work, Medina et al. [30] selected probiotics based on their ability to generate antibodies that cross-react with two pathogens (*Vibrio harveyi* and *Photobacterium damselae* subsp. *piscicida*) that are present in farmed Senegalese sole (*Solea senegalensis*). Among the probiotics used, the *Vibrio proteolyticus* DCF12.2 strain stood out for its great capacity to generate antibodies in fish. It also had other beneficial characteristics, such as its ability to inhibit pathogen growth, avirulence in fish, and survivability under storage conditions [30]. Furthermore, this species showed enzymatic activity, including the production of lecithinase, gelatinase, caseinase, amylase, and lipase, which can contribute to better aquafeed digestibility; continued viability after being added to the aquafeed; and survivability under the pH conditions found in the fish gut [31]. All the above features make this bacterium an excellent potential candidate for use in aquaculture.

The culture of Mugilidae species (mullets) is considered a priority within current European aquaculture strategies [32]. *Mugil cephalus* (Linnaeus, 1758), *Chelon ramada* (Risso, 1827), *Chelon labrosus* (Risso, 1827), and *Chelon saliens* (Risso, 1810) are the main Mugilidae fish species represented among the Mediterranean, each of which has great economic value [33]. One of the mullet species that can potentially be used in aquaculture is the thick-lipped grey mullet (*C. labrosus*). It is omnivorous in the early stages of development, becoming herbivorous with age [34]. Similar to other mullet species, *C. labrosus* has a high osmoregulatory capacity, allowing it to live in a wide range of salinities without compromising its development rate [35,36].

In a recent study by our group [37], we confirmed the suitability of using *C. fusca* as a dietary ingredient for *Chelon labrosus* juveniles. In the study, a diet containing 15% *C. fusca* enhanced the growth performance, nutrient utilization, flesh lipid quality, digestive and metabolic enzyme activity, absorptive capacity, and gut morphology of the fish.

The present study aimed to evaluate the dietary administration of *C. fusca* and ethanol-inactivated *V. proteolyticus* DCF12.2 in *C. labrosus* juveniles, highlighting the nutritional, physiological, and morphological effects. In addition, a comparative approach was also evidenced, in which *C. fusca* alone was included in the aquafeeds.

## 2. Materials and Methods

### 2.1. Ethical Approval

The study protocol was reviewed and approved by the Ethical Committee of the University of Malaga and the Andalusian Autonomous Government (Ref. 11/07/2020/082). The experimental protocol was performed according to the Guidelines of the European Union Council (2010/63/UE) and the use of laboratory animals (RD/1201/2005 and Law 32/2007).

### 2.2. Microalgae Production, Microorganism Inactivation, and Experimental Diets

The microalga *Chlorella fusca* was produced in pilot-scale photobioreactors (PBRs) at facilities that are part of the PILOT4U initiative at the University of Almeria (Spain). The biomass was produced in closed tubular photobioreactors (0.10 m tube diameter, 3 m^2^ volume, 80 m^2^ land surface), which had been previously inoculated with microalgae cultured in a bubble column (0.2 m diameter, 2.0 m height, 0.5 m land surface). The culture conditions inside the reactors were controlled, such as the pH, temperature, and dissolved oxygen (Crison Instruments, Barcelona, Spain). The pH was controlled by the on-demand injection of CO_2_. The temperature was controlled by passing hot/cold water through a heat exchanger installed inside the photobioreactor. Dissolved oxygen was controlled by modifying the airflow entering the reactor. The biomass was produced in irrigation water using the culture medium described by Sorokin and Krauss [38], prepared using commercial fertilizers. Filtration (0.02 μm) and ozonization (1 mg L^−1^) were performed to prevent contamination. To process the biomass, it was harvested by centrifugation (SSD6 GEA Westfalia, Oelde, Germany) and then subjected to cell disruption by high-pressure homogenization at 600 bars in a single pass (GEA Ariete NS3015H). Finally, to preserve the biomass, it was spray-dried at temperatures below 80 °C (GEA Mobile MinorTM Spray dryer). 

The bacterium *Vibrio proteolyticus* DCF12.2 was isolated from the intestine of healthy wedge sole (*Dicologlossa cuneata*). It was selected for this study because of its enzymatic activity and its ability to inhibit pathogen growth [27]. The *V. proteolyticus* DCF12.2 cells were cultured for 24 h at 22 °C on tryptic soy agar (TSA; Oxoid Ltd., Basingstoke, UK) and supplemented with NaCl (1.5%; TSAs). After incubation, the bacterial growth on the surface of all the plates was scraped off, suspended in sterile phosphate-buffered saline (PBS), and pooled. The cells were recovered by centrifugation (6000× *g*, 15 min, 4 °C) and the pellet was suspended in PBS, adjusted to 10^11^ colony forming units mL^−1^ (CFU mL^−1^) (O.D._600 nm_ = 1.0), and the viable cell concentration was determined by a plate count on TSA plates. The bacteria were inactivated with 70% ethanol and incubated at room temperature for 5 min [39]. Following the inactivation treatment, the number of viable bacteria was determined by plate counting on the TSAs to confirm the inactivation efficacy. Finally, the suspension was frozen at −80 °C for 12 h, and lyophilized using a LyoQuest (Telstar, Tokyo, Japan) laboratory freeze-drying system for 48 h at −85 °C. The lyophilized powder was stored in darkness at −20 °C until further preparation of the experimental diet. To verify that the enzymatic activity of all the cells described by Medina et al. [27] was maintained following inactivation, protease, gelatinase, lipase, and amylase activity were assayed according to Chabrillón et al. [40] on agar plates containing 2% *w*/*v* of skimmed milk (Pirinea, Pyrenees, Spain), 1% *w*/*v* of gelatin (Oxoid, Basingstoke, UK), 1% *w*/*v* of Tween-80 (Panreac, Barcelona, Spain), and 4% *w*/*v* of starch (Panreac, Spain), respectively. In all cases, 6-mm-diameter wells were made in the plates and filled with a suspension of *V. proteolyticus* in PBS (100 mg in 1 mL of PBS) and incubated at 23 °C for 24 h. The plates were monitored to check for the presence of a clear zone around the wells. Amylase activity was indicated by a clear zone around the wells after flooding the plates with Lugol. In addition, as a negative control, the wells were filled with PBS, while, as a positive control, the wells were filled with a suspension of *V. proteolyticus* cells (10^8^ ufc/mL). The absence of a clear zone was interpreted as no activity having occurred.

Two isonitrogenous and isolipidic experimental diets (40% and 7% on a dry matter basis, respectively) were formulated and elaborated by the Experimental Diets Service (CEIMAR, University of Almeria, Spain) using extrusion procedures to obtain 3 mm floating pellets. A diet was formulated that included 15% (*w*/*w*) of dry *C. fusca* biomass and 10^9^ cells kg^−1^ feed of the lyophilized *V. proteolyticus* DCF12.2 (diet C + V). In addition, a microalga- and bacteria-free diet was used as the control (CT). Table 1 and Table 2 summarize the formulations and fatty acid profiles of the experimental diets, respectively. Briefly, the feed ingredients were finely ground and mixed in a vertical helix ribbon mixer (Sammic BM-10, 10-L capacity, Sammic, Azpeitia, Spain) before the fish oil and diluted choline chloride were added to the blend. All the ingredients were mixed for 15 min, and water (350 mL per 1 kg of mass ingredient) was added to obtain a homogeneous dough. The dough was extruded using a two-screw extruder (Evolum 25, Clextral, Firminy, France). The extruder barrel consisted of four chambers that were maintained with a temperature profile (from inlet to the extruder die head) of 95 °C, 98 °C, 100 °C, and 110 °C, respectively. In the case of the C + V diet, the bacterial cells were applied using a vacuum lab mixer (Pegasus PG-10VC LAB, Dinnissen, Sevenum, The Netherlands) after the extrusion process. Finally, the pellets were dried at 27 °C in a drying chamber (Airfrio, Almeria, Spain) for 24 h, and kept in sealed plastic bags at −20 °C until use.

### 2.3. Fish Maintenance and Experimental Design

Thick-lipped grey mullet (*Chelon labrosus*) juveniles (*n* = 200) were provided by the Centro de Experimentación de Ecología y Microbiología de Sistemas Acuáticos Controlados Grice-Hutchinson (CEMSAC) of the University of Malaga (Malaga, Spain; Spanish Operational Code REGA ES290670002043) from a stock maintained in 5000 L tanks. Before beginning the trial, the fish were acclimatized to the experimental setup and fed a commercial diet (32% protein, 6% fat, TI-3 Tilapia, Skretting, Spain) for two weeks. Six homogeneous groups of 20 fish each (99.5 ± 0.2 g) were randomly distributed in six 1000 L tanks (~2 kg/m^3^) that were coupled to a recirculating aquaculture system (RAS) equipped with physical and biological filters, and maintained under a natural photoperiod for 90 days (April 2021–July 2021), within a temperature range of 19.7–22.9 °C and 1.0–1.2‰ salinity. Supplemental aeration was provided to maintain the dissolved oxygen at 6.8 ± 0.4 mg L^−1^. Ammonia (<0.1 mg L^−1^), nitrite (<0.1 mg L^−1^), and nitrate (<50 mg L^−1^) were determined weekly at 9:00 a.m. The two experimental dietary groups (CT and C + V) were then established in triplicate. The fish were manually fed twice a day (9:00 a.m. and 5:00 p.m.) at a rate of 1.5% of their body weight for 90 days. Feed rations were adjusted according to fish growth to maintain the initial 1.5% rate during the experiment. The wasted pellets were collected 60 min after administration, dried for 12 h at 120 °C, and then weighed.

### 2.4. Sample Collection

The fish were counted and group-weighed every 3 weeks, and the feed rations were adjusted accordingly to maintain the initial 1.5% of body mass rate during the experiment. At the end of the trial (day 90), overnight-fasted fish (3 fish per replicate, 9 per experimental group) were randomly selected and euthanized by immersion in water with a 2-phenoxyethanol overdose (1 mL L^−1^, Sigma-Aldrich 77699, St. Louis, MI, USA) [42], and then blood and tissue samples were collected. Blood was drawn from caudal vessels with heparinized syringes, centrifuged at 3000× *g* for 20 min at 4 °C, and the plasma samples were snap-frozen in liquid nitrogen and stored at −80 °C until further analysis. The livers were sampled and weighed immediately. Whole viscera were obtained, the intestines were separated from the other organs, and all visible perivisceral fat was removed. Liver samples and red and white muscle samples were taken and stored at −80 °C until biochemical analysis. All muscle was taken from a mediolateral location from the caudal peduncle. Specifically, the white muscle was found in the superficial layers, whereas the red muscle was closer to the spine. For the enzymatic analysis, the intestines from four fish per experimental tank were randomly pooled to obtain four different enzymatic extracts for each experimental tank (12 enzymatic extracts per dietary treatment). Intestinal samples from proximal and distal regions were homogenized in distilled water at 4 °C (*w*/*v* 1:2). Supernatants were obtained after centrifugation (13,000× *g*, 12 min, 4 °C) and stored at −20 °C until further analysis. Finally, 0.5 cm liver portions and 1 cm lengths of each intestinal region from three specimens per tank (9 fish per dietary treatment) were sampled for further evaluation under light microscopy (LM) and scanning electron microscopy (SEM).

### 2.5. Growth Performance and Somatic Indices

To analyze the growth performance, we evaluated the weight gain (WG), specific growth ratio (SGR), feed conversion ratio (FCR), protein efficiency ratio (PER), condition factor (K), and hepatosomatic index (HSI) using the following equations (Equations (1)–(6)):

WG (%) = ((final fish weight − initial fish weight) × 100)(1)

SGR (%) = (100 × [(ln final fish weight) − (ln initial fish weight)]/experimental days)(2)

FCR = dry feed intake (g)/weight gain (g)(3)

PER = weight gain/intake of dietary protein(4)

K (%) = ((fish weight/fish length^3^) × 100) (5)

HSI (%) = ((liver weight/body weight) × 100)(6)

### 2.6. Muscle Composition

Proximate composition analysis of the experimental diets and fish muscle samples was performed according to the AOAC [43] for dry matter and ash. The crude protein content (N × 6.25) was quantified using elemental analysis (C:H:N) (Fisons EA 1108 analyzer, Fisons Instruments, Waltham, MA, USA), while the total lipid content was obtained according to Folch et al. [44]. In addition, the fatty acid profile of the aquafeeds, liver, and muscle samples was determined by gas chromatography following the procedure described by Rodríguez-Ruiz et al. [45].

From the fatty acid profile of the fish muscle, the peroxidability index (PI), thrombogenicity index (IT), atherogenicity index (IA), and fish lipid quality (FLQ) were calculated [46,47] (Equations (7)–(10)):

PI = (% monoenoic × 0.025) + (% dienoic × 1) + (% trienoic × 2) + (% tetraenoic × 4) + (% pentaenoic × 6) + (% hexaenoic × 8)(7)

IT = (14:0 + 16:0 + 18:0)/[(0.5 × 18:1) + (0.5 × ΣMUFAs) + (0.5 × n-6 PUFAs) + (3 × n-3 PUFAs) + (n-3/n-6) (8)

IA = (12:0 + 4 × 14:0 + 16:0)/[(n-6 + n-3) PUFAs + 18:1 + other MUFAs];(9)

FLQ (%) = [(20:5n3 + 22:6n3)/total lipid] × 100(10)

MUFAs and PUFAs stand for monounsaturated fatty acids and polyunsaturated fatty acids.

### 2.7. Tissue Metabolites

Tissue metabolite levels were analyzed from the liver samples and the red and white muscle samples (three fish per tank, 9 per experimental group). The samples were individually minced on an ice-cold Petri dish and then homogenized by mechanical disruption (Ultra-Turrax^®^, T25basic, with an S25N-8G dispersing tool, IKA^®^-Werke, Stauffen, Germany) with 7.5 vol. (*w*/*v*) of ice-cold 0.6 N perchloric acid and neutralized after adding the same volume of 1M KHCO_3_. Subsequently, the homogenates were centrifuged (3500× *g*, 30 min, 4 °C), and the supernatants were recovered in different aliquots. The aliquots were then stored at −80 °C until use in the metabolite assays. 

The metabolite concentrations (glucose, lactate, and triglycerides) in the plasma and liver were determined using commercial kits from Spinreact (Barcelona, Spain) (Glucose-HK Ref. 1001200; Lactate Ref. 1001330; Triglycerides Ref. 1001311) with the reactions adapted to 96-well microplates. Hepatic glycogen levels were assessed using the method of Keppler and Decker [48]. After subtracting the free glucose levels, the glucose obtained after glycogen was determined using the commercial glucose kit described above. All the standards and samples were measured in duplicate. All the assays were run on an Automated Microplate Reader (PowerWave 340, BioTek Instrument Inc., Winooski, VT, USA) using KCjunior™ software.

### 2.8. Activity of Metabolic Enzymes in the Liver

Frozen liver biopsies (three fish per tank, 9 per experimental group) used for the enzymatic activity assays were homogenized by mechanical disruption (Ultra-Turrax^®^, Staufen, Germany) with 10 vol. (*w*/*v*) of ice-cold homogenization buffer (50 mM imidazole, 1 mM 2-mercaptoethanol, 50 mM NaF, 4 mM EDTA, 0.5 mM phenylmethylsulfonyl fluoride (PMSF), and 250 mM sucrose; pH 7.5). The homogenates were centrifuged for 30 min at 3220× *g* and 4 °C, and the supernatants were stored at −80 °C for further analysis. The assays to determine several enzymes involved in glycogenolysis (GPase (active): glycogen phosphorylase, EC 2.4.1.1), glycolysis (HK: hexokinase, EC 2.7.1.1; PK: pyruvate kinase, EC 2.7.1.40), gluconeogenesis (LDH: lactate dehydrogenase, EC 1.1.1.27; FBP: fructose 1,6-bisphosphatase, EC 3.1.3.11), and lipid metabolism (HOAD: 3-hydroxyacyl-CoA dehydrogenase, EC 1.1.1.35) were performed as previously described for gilthead seabream (*Sparus aurata*) tissues [49]. All the assays were run on an Automated Microplate Reader (PowerWave 340, BioTek Instrument Inc., Winooski, VT, USA) using KCjunior™ software (BioTek Instrument Inc.). Activity was expressed as specific activity per mg of protein in the homogenate (U mg prot^−1^). Proteins were assayed in duplicate with the bicinchoninic acid method using a commercial BCA kit (BCA™ Protein assay kit, Pierce, Rockford, IL, USA).

### 2.9. Determination of Digestive Enzyme Activity

Total alkaline protease activity was determined at 280 nm using buffered 5 g L^−1^ casein (50 mM Tris-HCl, pH 9.0) as the substrate according to Alarcón et al. [50]. One unit of activity (UA) was defined as the amount of enzyme releasing 1 μg tyrosine per minute (an extinction coefficient for tyrosine of 0.008 mg^−1^ cm^−1^ mL^−1^). The activity of trypsin and chymotrypsin was quantified using 0.5 mM (BAPNA) (N-a-benzoyl-DL-arginine-4-nitroanilide) and 0.2 mM SAPNA (N-succinyl-(Ala)2-Pro-Phe-Pnitroanilide) in 50 mM Tris–HCl buffer, pH 8.5, containing 10 mM CaCl_2_, according to Erlanger et al. [51] and DelMar et al. [52], respectively. Leucine aminopeptidase activity was determined using buffered 2 mM L-leucine-p-nitroanilide (LpNa) (100 mM Tris–HCl, pH 8.8) as the substrate according to Pfleiderer [53]. The alkaline phosphatase activity was quantified using buffered p-nitrophenyl phosphate (pH 9.5) as the substrate [54]. One unit of activity of trypsin, chymotrypsin, and leucine aminopeptidase was defined as the amount of enzyme that released 1 μmol of p-nitroanilide (pNA) per minute (an extinction coefficient of 8800 M cm^−1^ at 405 nm). For alkaline phosphatase, one UA was established as the amount of enzyme that released 1 μg of nitrophenyl per min (an extinction coefficient of 17,800 M cm^−1^ at 405 nm). All the assays were performed in triplicate and the specific enzyme activity was expressed as U g tissue^−1^.

### 2.10. Liver and Intestine Histological Analysis

The liver and intestine samples were fixed for 24 h in phosphate-buffered formalin (4% *v*/*v*, pH 7.2), dehydrated, and embedded in paraffin in accordance with standard histological techniques [55]. Briefly, the samples were placed so that the intestines and liver were cut into 5 μm transversal sections. All the slides were stained with hematoxylin–eosin (H&E). The stained preparations were examined under a light microscope (Olympus ix51, Olympus, Barcelona, Spain) equipped with a digital camera (CC12, Olympus Soft Imaging Solutions GmbH, Muenster, Germany) and the images were analyzed with specific software (Image J, National Institutes of Health, Bethesda, MD, USA). The hepatocyte area and hepatocyte major axis were measured in the liver samples, whereas, in intestinal samples, the length and diameter of the mucosal folds, the total enterocyte height, and the thickness of the lamina propria of the submucosa, muscular, and serous layer were analyzed. At least 15 independent measurements per animal were obtained.

### 2.11. Ultrastructural Analysis of the Intestinal Mucosa

The scanning electron microscopy (SEM) samples were processed as described in Vizcaíno et al. [55]. The intestinal samples were fixed for 24 h in phosphate-buffered formalin (4% *v*/*v*, pH 7.2). Then, the fixed samples were washed with phosphate buffer and progressively dehydrated in graded ethanol. After this, the samples were dried in a critical point dryer (CDP 030 Critical Point Dryer, Leica Microsystems, Madrid, Spain) using absolute ethanol as the intermediate fluid and CO_2_ as the transition fluid. The dried samples were mounted on supports, fixed with graphite (PELCO^®^ Colloidal Graphite, Ted Pella Inc., Redding, CA, USA), and gold sputter-coated (SCD 005 Sputter Coater, Leica Microsystems). All the samples were screened via high-resolution field emission scanning electron microscopy (FESEM) (Carl Zeiss, Sigma 300 VP, Jena, Germany). All the digital images were analyzed with Image J (National Institutes of Health, USA) software and a morphometric analysis was carried out to determine the enterocyte apical area (EA) [55].

### 2.12. Statistical Analysis

All the statistical analyses were performed with SPSS Statistics 26 software. Results are reported as means ± SEM (*n* = 9). Normal distribution was checked for all the data with the Shapiro–Wilk test, while the homogeneity of the variances was obtained using the Levene test. When necessary, an arcsine transformation was performed. Differences between the two experimental diets (CT and C + V) were tested using Student’s *t*-test. Two-way ANOVA was applied to determine statistical differences between the metabolite content in the white and red muscle according to the diets. In all the statistical tests performed, *p* < 0.05 was considered significantly different.

Furthermore, in order to elucidate whether the positive effects found in this work were attributable to the microalgae alone or the combined use of the microalgae and the inactivated bacteria, we conducted two different analyses. First, Student’s *t*-test was used to determine if there were differences in the *Chelon labrosus* fed with the 15% *C. fusca* (C-15) diet compared to its control diet (CT EXP1) (García-Márquez et al. [37]) and specimens fed with the combination of *C. fusca* and *V. proteolyticus* (C + V) compared to its control diet (CT EXP2) (the present study) in terms of growth performance and nutrient utilization ratios. For this, we used the mean of each independent replicate tank against the mean of the controls (the C-15/CT EXP1 and C + V/CT EXP2 quotients) in each parameter. Second, a one-way ANOVA was applied to determine statistical differences in the EPA + DHA content in fish fillets from wild specimens (García-Márquez et al. [56]), from specimens fed the 15% *C. fusca* (C-15) diet compared to its control diet (CT EXP1) (García-Márquez et al. [37]), and from specimens fed with the combination of *C. fusca* and *V. proteolyticus* (C + V) compared to its control diet (CT EXP2) (the present study).

## 3. Results

### 3.1. Enzymatic Activity of the Inactivated Bacteria

After inactivation with ethanol, the bacteria’s absence of growth in TSAs was verified. Nevertheless, enzymatic activity (caseinase, gelatinase, lipase, and amylase) was maintained after the bacteria’s ethanol inactivation.

### 3.2. Growth Performance and Biometric Parameters

No mortality occurred during the experiment. Diet C + V statistically improved the growth performance in *C. labrosus* juveniles (Table 3). After 90 days, the body weight, weight gain, Fulton’s Condition Factor, and protein efficiency were all significantly higher in fish fed the C + V diet compared to the CT group. Furthermore, the feed conversion ratio was statistically lower in the fish fed the C + V diet than in the CT group. No significant differences were found in the length, specific growth rate, or hepatosomatic index.

### 3.3. Muscle Composition and Fatty Acid Profile

The muscle proximate composition is shown in Table 4. No significant differences were observed for protein, total lipids, and ash when the fish were fed the microalgae and *V. proteolyticus* supplemented diets (*p* > 0.05).

The muscle fatty acid profiles are shown in Table 5, and there were differences between the experimental groups. Oleic acid (18:1n9) and palmitic acid (16:0) were the most abundant fatty acids in both groups. In muscle, the CT group had significantly higher proportions of myristic acid (14:0) and oleic acid. On the other hand, the C + V specimens presented statistically higher proportions of arachidonic acid (ARA, 20:4n6), eicosapentaenoic acid (EPA, 20:5n3), and docosahexaenoic acid (DHA, 22:6n3). Total saturated and polyunsaturated fatty acids (SFA and PUFA, respectively) were not modified by the experimental diet. In contrast, while a significantly higher proportion of monounsaturated fatty acids (MUFA) and n-9 was found in the muscle of the control specimens, a higher n-3 and n-3/n-6 ratio was detected in the C + V samples. As a result, the C + V specimens showed a significantly higher peroxidability index (PI) and fish lipid quality (FLQ), and a lower index of thrombogenicity (IT). 

### 3.4. Tissue Metabolites

The plasma and liver metabolites are shown in Table 6. A statistical increase in the plasma glucose and triglyceride values was observed in the C + V group compared to the CT group. However, the experimental diets did not cause significant variations in plasma lactate values. In the liver, glycogen levels decreased statistically in the C + V group with respect to the CT group, while triglyceride and lactate levels increased significantly. No significant differences were detected in hepatic free glucose among the experimental groups.

Glucose, glycogen, and triglycerides were significantly higher in red muscle than in white muscle, whereas white muscle had higher lactate content (Table 7). Regarding the dietary effects, dietary supplementation did not modify the values of any metabolite in the white muscle of either group. In contrast, significantly higher lactate content and lower glycogen values were found in the red muscle of fish fed the C + V diet compared to fish fed the CT diet.

### 3.5. Metabolic Enzymes

**Figure 1:**
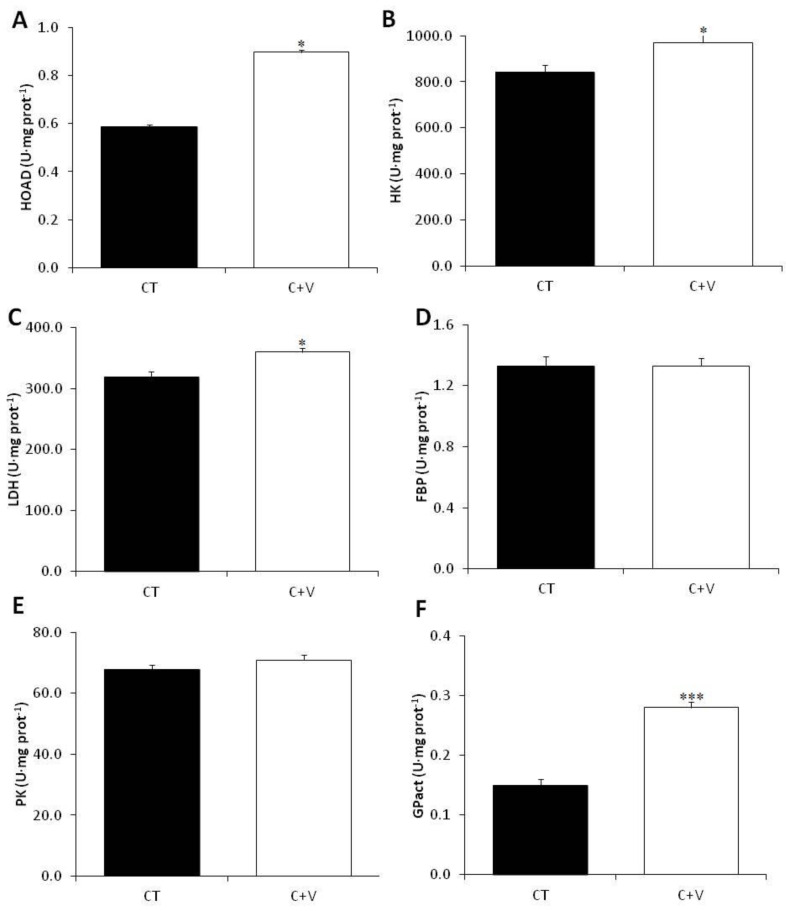
Specific activity (U mg protein^−1^ as mean ± SEM) of metabolic enzymes in the liver of juvenile *C. labrosus* fed the control (CT) and *C. fusca* + *V. proteolyticus* (C + V) diets for 90 days. Asterisks denote significant differences at *p* < 0.05 (*), *p* < 0.01 (**), and *p* < 0.001 (***). (**A**) HOAD: 3−hydroxyacyl−CoA dehydrogenase; (**B**) HK: hexokinase; (**C**) LDH: lactate dehydrogenase; (**D**) FBP: fructose 1,6−bisphosphatase; (**E**) PK: pyruvate kinase; (**F**) GPase: glycogen phosphorylase (active).

Figure 1 shows the results for the metabolic enzymes related to glycogenolysis, glycolysis, gluconeogenesis, and lipid metabolism in the liver. HOAD (lipid metabolism), HK (glycolysis), LDH (gluconeogenesis), and GPact (glycogenolysis) activity significantly increased in the C + V group. No significant effects were detected in either PK (glycolysis) or FBP (gluconeogenesis) enzymatic activity.

### 3.6. Digestive Enzyme Activity

The results for the digestive enzyme activity analysis are summarized in Table 8. Overall, the inclusion of *C. fusca* and *V. proteolyticus* did not negatively affect any of the digestive enzyme studies. Fish fed the C + V diet had significantly increased total proteolytic activity in the proximal and distal intestines compared to fish fed the CT diet (*p* < 0.05). Chymotrypsin activity was similar in both groups, while trypsin and total alkaline protease were enhanced significantly in fish fed the C + V diet. Regarding intestinal brush enzymes, leucine aminopeptidase and alkaline phosphatase activity levels were significantly higher in fish fed the microalgae and *V. proteolyticus*-supplemented diet compared to the CT group in both the proximal and distal intestinal regions (*p* < 0.05).

### 3.7. Histological Evaluation of the Liver and Intestinal Mucosa

**Figure 2:**
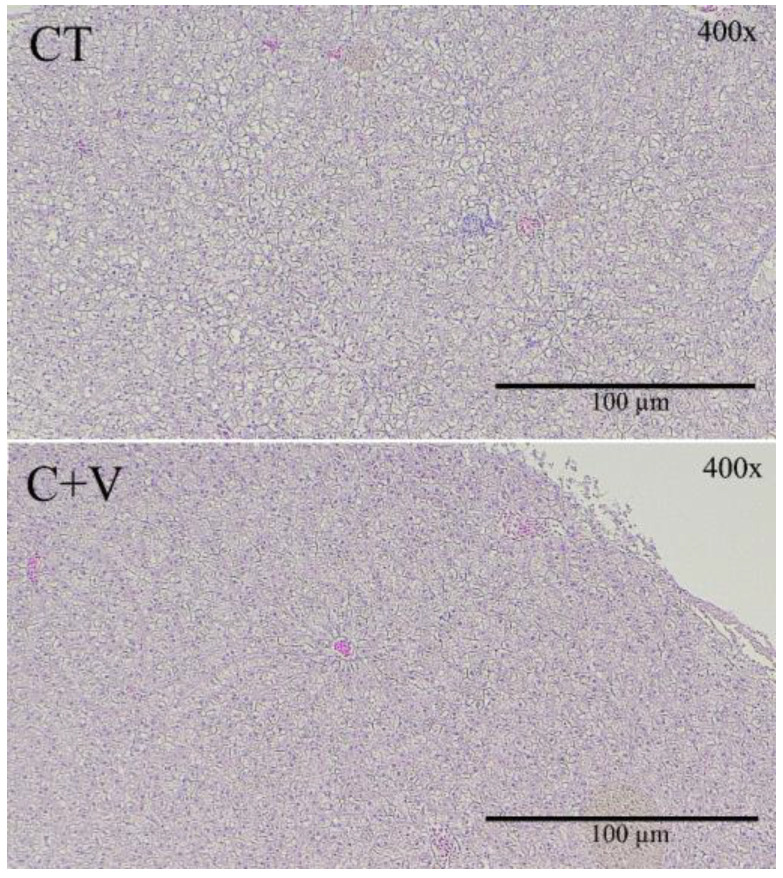
Liver light microscopy details of juvenile *C. labrosus* fed the control (CT) or *C. fusca* + *V. proteolyticus* (C + V) diets for 90 days. Scale bar: 200 μm.

No evidence of severe necrosis or steatosis was found since all the fish exhibited normal-shaped hepatocytes with regular morphology (Figure 2). In addition, the morphometrical evaluation revealed the absence of any significant difference in hepatocyte apical area or hepatocyte major axis between the dietary treatments (Table S1).

**Figure 3:**
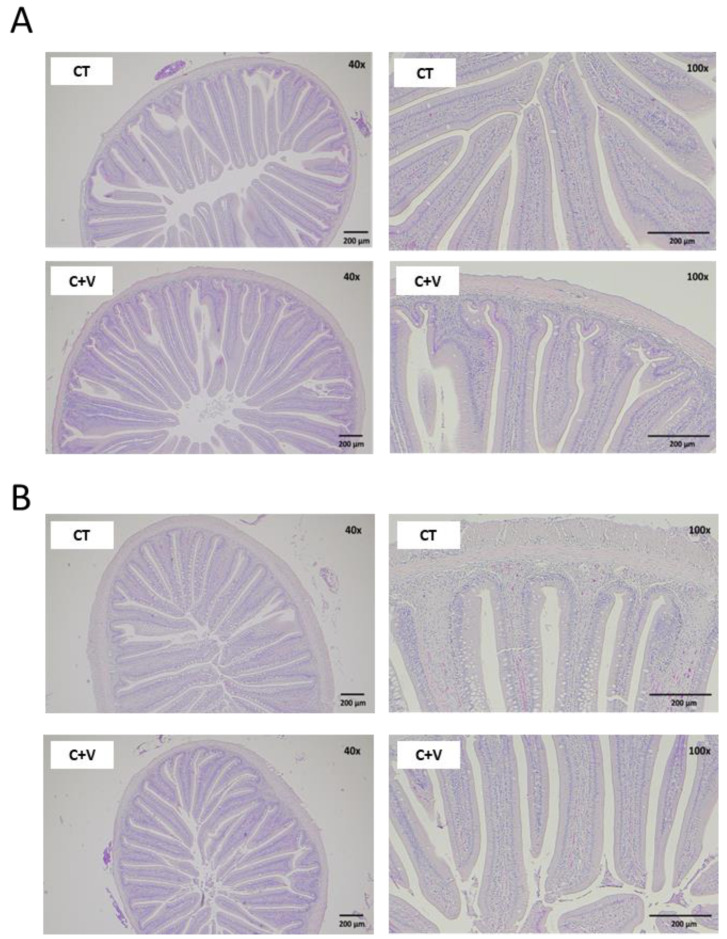
Light microscopy images from the proximal (**A**) and distal (**B**) regions of the intestine of juvenile *C. labrosus* fed the control (CT) or *C. fusca* + *V. proteolyticus* (C + V) diets for 90 days. H&E stain, magnification 40×, scale bar 200 μm; 100×, scale bar 200 μm.

Furthermore, no histological modifications suggesting intestinal damage attributable to the dietary treatments were found in the intestinal preparations (Figure 3). Nevertheless, moderate enterocyte vacuolization was observed in several samples from fish fed the CT diet, especially in the distal region (Figure 3B).

The morphometric analysis (Table 9) revealed a significant effect induced by microalgae and *V. proteolyticus* inclusion in both the proximal and distal intestinal regions. In general, the length of the intestinal folds in the proximal region was significantly greater (*p* < 0.05) in fish fed the C + V diet, while remaining unaffected in the distal intestine. The fold diameter was greater in the CT group regardless of the region evaluated. The C + V diet reduced the thickness of the serosa and submucosa layers, and the lamina propria, compared to the fish fed the CT diet (*p* < 0.05).

### 3.8. Ultrastructural Analysis of Intestinal Mucosa

**Figure 4:**
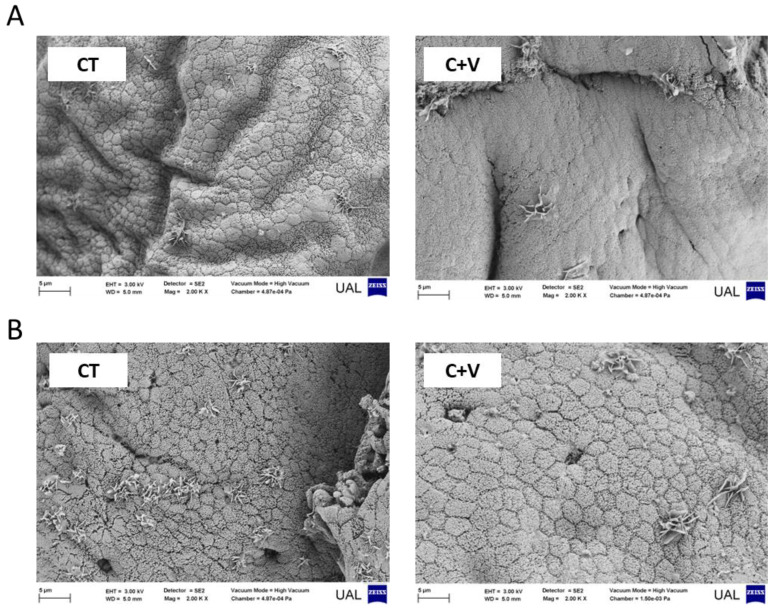
Comparative SEM micrographs from the proximal (**A**) and distal (**B**) intestinal regions of juvenile *C. labrosus* fed the control (CT) or *C. fusca* + *V. proteolyticus* (C + V) diets for 90 days. (bar: 5 µm).

**Figure 5:**
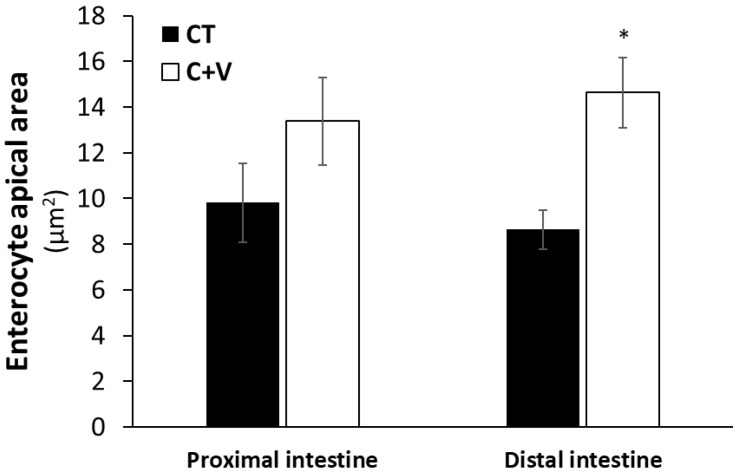
Enterocyte apical area (µm^2^) of the proximal and distal intestinal regions of juvenile *C. labrosus* fed the control (CT) or *C. fusca* + *V. proteolyticus* (C + V) diets for 90 days. Values are expressed as mean ± SEM (*n* = 50 measurements per treatment). Asterisks denote significant differences (*p* < 0.05) between experimental diets in the same intestinal region.

The SEM observations confirmed the integrity of the enterocytes in the fish fed both experimental diets. The intestines presented a mucosa of normal appearance with no intercellular spaces in the apical zone of the epithelium (Figure 4). A morphometric analysis of the SEM images revealed a significant increase (*p* < 0.05) in the enterocyte apical area in fish fed the C + V diet compared to the CT group (Figure 5).

## 4. Discussion

Microalgae and bacteria have been assessed separately as functional ingredients in feed for various fish species. However, little is known about the effects of combining them in aquaculture species. Studies on the combined effect of microalgae and probiotics mainly focus on immune responses, gene expression, intestinal morphology, and the microbiota [57,58,59].

The results of the present study have shown that the combined inclusion of *C. fusca* and *V. proteolyticus* in *C. labrosus* diets significantly promoted growth performance and feed utilization compared to the control group (a non-supplemented diet). High growth performance was also reported by Reyes-Becerril et al. [60] after 8 weeks of feeding a combination of the microalgae *Navicula* sp. and the probiotic *Lactobacillus sakei* to Pacific yellow snapper (*Lutjanus peru*). Our research group recently investigated the effects of *C. fusca* in *C. labrosus* diets, finding increased growth performance in the fish receiving the microalgae-supplemented diet [37]. In this regard, mixing microalgae and bacteria enhanced feed efficiency (FCR and PER) and the fish condition factor, which remained unaltered when *C. fusca* was incorporated separately in aquafeeds [37]. This difference may be due to enzymatic activity (lecithinase, gelatinase, caseinase, amylase, and lipase) in *V. proteolyticus* whole cells [30], which continues after ethanol inactivation. This activity might allow the host to better digest the aquafeed and improve the availability of nutrients. This agrees with the study by De Schrijver and Ollevier [61], which reported that *V. proteolyticus* ingestion stimulates protein digestibility in turbot (*Scophthalmus maximus*).

Muscle proximate composition is considered an indicator of the physiological and health status of fish [62]. Despite the fact that none of the dietary treatments tested in this work modified the muscle composition, it is known that the inclusion of probiotic microorganisms and microalgae in aquafeeds may exert a significant effect on the chemical composition of the animal. Authors such as Pandey et al. [63] observed an increase in muscle protein content in common carp (*Cyprinus carpio*) specimens fed *Lactobacillus plantarum*-supplemented diets, which seems to be associated with improved feed efficiency rates. Meanwhile, microalgae inclusion is related to fish metabolism, especially with lipids used as an energy source, which lead to a reduction in lipid tissue storage [64].

Regarding the fatty acid composition, dietary changes are reflected in the fatty acid composition of marine fish tissues [65]. As expected, the fatty acid profiles of experimental feeds were reflected in an increased proportion of 16:0, 18:1n9, and 18:2n6 in the muscle of both groups. Furthermore, the C + V diet induced a selective retention of ARA, EPA, and DHA in the muscle, which agrees with findings from other marine fish species fed with algae-supplemented diets [66,67,68]. This points to a relationship between the algae inclusion level and the higher efficiency of mobilizing lipids. Inadequate intake of these fatty acids, which are essential for the cellular membrane structure and function, results in decreased growth and increased fish mortality, as well as other pathologies, such as liver or intestinal steatosis [69]. The relative increase in structural fatty acids (ARA, EPA, and DHA) in the muscle is also reflected in the significant increase in the fish lipid quality index (FLQ), n-3 polyunsaturated fatty acid, and n-3/n-6 ratio in the fish fed the C + V diet, which could be beneficial from a human nutrition standpoint [70,71]. 

The metabolic response to the experimental diets was also investigated. The C-V diet considerably activated the carbohydrate metabolism by statistically boosting the plasma glucose and lowering (not statistically) the plasma lactate. Thus, we propose that the C + V diet may promote plasma lactate depletion, which originates in the white muscle due to anaerobic metabolism and is then partially incorporated in the liver; this is consistent with the findings of other authors [37,49]. Furthermore, a significant increase in LDH activity was found in fish fed the C + V diet; this is consistent with prior research in which gilthead seabream were fed diets enriched with microalgae [72]. This increase in LDH activity appears to indicate the conversion of lactate to pyruvate via the Cori cycle, which may then be converted and stored as glycogen or for energy within the liver or elsewhere.

Hepatic TAG levels increased in the C + V fed fish compared to those fed the control diets. The liver is the primary site of lipogenesis in fish, and hepatic carbohydrates may regulate the total lipogenesis [73]. Because both metabolites, glycogen and TAG, served as energy reserves, our findings imply that more energy is invested in maintenance and development than in storage, which is consistent with the biometric data obtained, and those reported by other authors [72].

It was also shown that fish fed the C + V diet had significantly increased hepatic HK activity. Nevertheless, the PK activity remained unchanged, which is consistent with the findings of Perera et al. [49] and our earlier work evaluating *C. fusca* as a dietary ingredient for *C. labrosus* [37]. HK catalyzes the initial stage of glycolysis, phosphorylating glucose so that it can be used by the cells, whereas PK catalyzes the last step, creating pyruvate and ATP. Interestingly, Perera et al. [49] found that 1% microalgae supplementation increased the hepatocyte capacity for glucose uptake and glycogen storage but not the oxidation for energy; this differs from the findings of Molina-Roque et al. [72], who found that higher inclusion (10%) in identical diets to those used by Perera et al. [49] increased the oxidation for energy. Although our work and the studies mentioned above were conducted in two different species, it appears that the inclusion of microalgae-derived products in aquafeeds consistently promotes glucose uptake by hepatocytes. Nonetheless, the final fate (i.e., storage or oxidation) varies depending on the level of microalgae inclusion. Furthermore, the studies by García-Márquez et al. [37] and Molina-Roque et al. [72] showed a decrease in hepatic gluconeogenesis-related FBP enzyme activity [74], supporting the glucose oxidation hypothesis. In our research, however, the hepatic FBP levels were unchanged.

Our results also show a significant increase in GPase activity in fish fed the C + V diet. Using glycogen as a substrate, this enzyme catalyzes the dephosphorylation of glucose-6-phosphate to glucose [75]. Thus, the increased enzyme activity correlates well with the reduction in hepatic glycogen reported in the C + V-fed fish. Lastly, fish fed the C + V diet had significantly increased HOAD activity. The HOAD enzyme is involved in fatty acid β-oxidation [76], and its activity is directly related to lipid availability [77]. This finding may be linked to the observed increased in hepatic glucose oxidation for energy, which would enhance triglyceride levels in the liver, making them more accessible to promote muscle development, as postulated by Molina-Roque et al. [72].

On the other hand, the evaluation of digestive enzymes and the knowledge of their involvement in the digestion and absorption processes are key tools that can be used as a reliable indicator of the nutritional status of aquaculture fish, as well as to select new ingredients to use in aquafeed production [50]. Previous works have shown that using both probiotic microorganisms and microalgae induces significant changes in some of the enzymes involved in digestion and absorption processes, increasing the activity levels of both pancreatic and brush border secretion enzymes [14,37,55]. Overall, the results from the assays in this study indicate that the dietary inclusion of *C. fusca* together with *V. proteolylicus* increased the enzyme activity assayed in fish fed the control diet. It is widely known that higher secretion of digestive enzymes induces an improvement in protein digestion, which contributes to better feed utilization [55]. This could explain the positive effects observed on growth and the feed utilization rate derived from the inclusion of microalgae and probiotics in feed. 

It is also worth mentioning the favorable effects of the *C. fusca* + *V. proteolyticus*-supplemented diet on brush border digestive activity (leucine aminopeptidase and alkaline phosphatase) that we observed in this work. These enzymes, especially alkaline phosphatase, a dominant enzyme in the intestinal brush border, can be used as an indicator of intestinal integrity and nutrient absorption [78,79]. Indeed, these positive effects concur with the histological and ultrastructural determinations of the intestinal mucosa.

A healthy gut is essential for optimal animal performance [55,80], so any alteration in the intestinal mucosa’s integrity strongly activates immune cells and may cause chronic inflammation of the intestinal tissue. There is evidence to confirm that the use of probiotic microorganisms and microalgae in aquafeeds seems to have a positive effect on the gut morphology of various fish species [68,81,82]. In our study, although an increase in the mucosal fold length was observed in the fish fed the *C. fusca* and *V. proteolyticus*-supplemented diet, a significant reduction in the thickness of the serosa and submucosa layer was also observed in comparison to the control group. It is important to note that the dietary inclusion of microalgae and probiotics induced a marked reduction in the level of vacuolization observed in the distal intestine of fish fed the control diet, as well as in the thickness of the lamina propria. In this regard, some studies carried out on zebrafish pointed out that certain microalgae (*Tetraselmis* sp.; *Phaeodactylum tricornutum*; *Chlorella* sp.; *Nannochloropsis oculata* or *Nannochloropsis gaditana*) and probiotic microorganisms are efficient in reducing the intestinal inflammatory response [83,84]. 

Similarly, the scanning electron microscopy analysis showed no signs of damage in the apical brush border. In agreement with that observed in previous works carried out on a variety of marine fish species (gilthead seabream, Senegalese sole, thick-lipped grey mullet) at different developmental stages (fry and juvenile) [37,64,68], the inclusion of microalgae in aquafeeds exerted positive changes in the enterocyte apical area, which can translate into an overall increase in the enterocyte absorption surface and, consequently, an enhanced intestinal absorption capacity [55]. 

In order to elucidate whether the positive effects found in this work are attributable to the microalgae alone or the combined use of the microalgae and the inactivated bacteria, we compared the growth performance, nutrient utilization, and the n-3 long-chain polyunsaturated fatty acids of the muscle samples from the current experiment with the results obtained by García-Márquez et al. [37]. The results showed that there were no differences between the two experimental diets (C-15 from García-Márquez et al. [37] and C + V from the present study) (Table S2, Figure S1), despite the fact that the experiments were carried out on fish of different sizes and over different photoperiods. Although the values of polyunsaturated fatty acids in the experimental diets were not as high as the values obtained in wild fish (García-Márquez et al. [56]), they were significantly higher than in fish fed the control feeds. This demonstrates that the use of microalgae in the diet can be a good strategy for increasing the polyunsaturated fatty acids in the flesh of farmed fish, bearing in mind that these fatty acids are important for human nutrition. In this regard, the Food and Agriculture Organization (FAO), and the European Food Safety Authority (EFSA) recommend a daily EPA and DHA intake of at least 250 mg [85,86]. Both the diets that included microalgae (C-15 from García-Márquez et al. [37] and C + V from the present study) were higher than the reference value for good health, indicating that the specimens can be considered a good source of EPA and DHA in human diets. 

The similar values obtained with both diets show that the inclusion of the inactivated bacteria did not affect the growth and lipid composition of the fish. However, the inclusion of inactivated bacteria in the experimental diet may have affected the enterocyte morphology and the disappearance of vacuoles in the enterocytes compared to fish fed the control diet. In this sense, García de La Banda et al. [87] observed that the inclusion of the probiotic *Shewanella putrefaciens* pdp11 in the diet of *Solea senegalensis* caused the removal of the lipid droplets inside the enterocytes of those fish compared to fish fed the control feed.

## 5. Conclusions

In summary, feed supplemented with *C. fusca* and *V. proteolyticus* has been shown to be suitable for feeding *Chelon labrosus*, increasing their growth performance and the nutritional characteristics of their muscle and intestine, without causing tissue damage.

## Figures and Tables

**Table 1:** Ingredient composition of the experimental diets.

	Control	C + V
Ingredients (g kg^−1^ dry weight, DW)		
Fish meal LT94 ^1^	75	64
*Chlorella fusca* biomass *^2^*		150
Pea protein concentrate ^3^	75	64
Soybean protein concentrate ^4^	175	149
Soybean meal	188	159
Sunflower meal	127	108
Wheat gluten ^5^	60	51
Wheat meal ^6^	210	170
Potato starch	25	25
Fish oil	40	35
Vit and Min premix ^7^	10	10
Binder	15	15
Chemical composition (g kg^−1^ dry weight, DW)		
Crude protein	394.2	381.7
Crude lipid	79.1	72.6
Ash	130.3	142.1
Nitrogen-free extracts ^8^	396.4	403.6
Gross energy (MJ/kg) ^9^	19.0	18.6

Dietary codes: CT, control diet; C + V, *C. fusca* + *V. proteolyticus* supplemented diet. DW: dry weight. ^1^ (Protein content, PC: 69.4%; lipid content, LC: 12.3%), Norsildemel (Bergen, Norway); ^2^ (PC: 15.2%; LC: 1.1%); ^3^ (PC: 85.5%; LC: 1.3%); ^4^ (PC: 51.5%; LC: 8.0%); ^5^ (PC: 76.0%; LC: 1.9%); ^6^ (PC: 12.0%; LC: 2.0%); ^7^ Vitamin and mineral premix: Vitamins (IU or mg kg^−1^ premix): vitamin A (retinyl acetate), 2000,000 IU; vitamin D3 (DL-cholecalciferol), 200,000 IU; vitamin E, 10,000 mg; vitamin K3 (menadione sodium bisulfite), 2500 mg; vitamin B1 (thiamine hydrochloride), 3000 mg; vitamin B2 (riboflavin), 3000 mg; calcium pantothenate, 10,000 mg; nicotinic acid, 20,000 mg; vitamin B6 (pyridoxine hydrochloride), 2000 mg; vitamin B9 (folic acid), 1500 mg; vitamin B12 (cyanocobalamin), 10 mg; vitamin H (biotin), 300 mg; inositol, 50,000 mg; betaine, 50,000 mg; vitamin C (ascorbic acid), 50,000 mg. Minerals (mg kg^−1^ premix): Co (cobalt carbonate), 65 mg; Cu (cupric sulfate), 900 mg; Fe (iron sulfate), 600 mg; I (potassium iodide), 50 mg; Mn (manganese oxide), 960 mg; Se (sodium selenite), 1 mg; Zn (zinc sulfate) 750 mg; Ca (calcium carbonate), 186,000 mg; KCl, 24,100 mg; NaCl 40,000 mg; excipient sepiolite, colloidal silica (Lifebioencapsulation SL, Almería Spain). ^8^ Calculated as: 100 − (% crude protein + % ether extract + % ash). ^9^ Gross energy was estimated by energetic coefficients (kJ/g) according to Miglavs and Jobling [41]: crude protein, 23.6; crude lipid, 38.9; Nfe, 16.7.

**Table 2:** Fatty acid composition (% of total fatty acids) of the experimental diets.

Fatty Acids	Experimental Diets	
	CT	C + V
14:0	3.19	2.81
16:0	22.30	21.34
16:1n7	4.63	4.23
16:2n4	0.91	0.61
16:3n4	0.57	0.57
18:0	6.04	5.18
18:1n7	2.04	1.99
18:1n9	17.13	14.81
18:2n6	14.79	14.96
18:3n3	1.21	3.01
18:4n3	0.60	0.61
20:1n9	2.17	1.71
20:4n6	0.49	0.53
20:5n3	3.92	4.71
22:5n3	2.21	1.12
22:6n3	10.18	11.80
SFA	31.53	29.34
MUFA	25.97	22.74
PUFA	34.56	37.97
Other FA	6.46	8.77
n-3	19.28	22.48
n-6	15.28	15.49
n-9	4.21	3.70
n-3/n-6	1.26	1.45
EPA/DHA	0.39	0.40

Dietary codes: CT, control diet; C + V, *C. fusca* + *V. proteolyticus* supplemented diet. EPA: eicosapentaenoic acid; DHA: docosahexaenoic acid.

**Table 3:** Growth performance and somatic indices of juvenile *C. labrosus* fed the control (CT) and *C. fusca* + *V. proteolyticus* (C + V) diets for 90 days.

Parameters	CT	C + V	*p*
Initial weight (g)	99.35 ± 0.09	99.86 ± 0.09	0.689
Final weight (g)	145.44 ± 0.22	152.45 ± 0.11 *	0.004
Initial length (cm)	19.16 ± 0.02	19.10 ± 0.03	0.456
Final length (cm)	21.69 ± 0.01	21.84 ± 0.04	0.534
WG (%)	146.38 ± 0.00	152.68 ± 0.02 *	0.013
SGR (%)	0.42 ± 0.01	0.47 ± 0.03	0.198
FCR	3.90 ± 0.21	3.12 ± 0.17 *	0.032
PER	0.66 ± 0.04	0.98 ± 0.12 *	0.029
K	1.40 ± 0.01	1.43 ± 0.01 *	0.046
HSI (%)	0.94 ± 0.06	0.97 ± 0.05	0.401

Dietary codes: CT, control diet; C + V, *C. fusca* + *V. proteolyticus* supplemented diet. Data on feed intake and growth indices are expressed as mean ± SEM (standard error of the mean) for triplicate tanks. Data on the hepatosomatic index are the mean ± SEM of 9 fish per treatment. Asterisks denote significant differences (*p* < 0.05).

**Table 4:** Muscle proximate composition (% dry weight) of juvenile *C. labrosus* fed the control (CT) and *C. fusca* + *V. proteolyticus* (C + V) diets for 90 days.

	CT	C + V	*p*
Protein	78.45 ± 0.30	79.39 ± 0.37	0.637
Total lipid	10.90 ± 0.10	10.41 ± 0.29	0.136
Ash	4.97 ± 0.05	5.07 ± 0.02	0.206

Dietary codes: CT, control diet; C + V, *C. fusca* + *V. proteolyticus* supplemented diet. Values are expressed as mean ± SEM (standard error of the mean) of 9 fish per treatment.

**Table 5:** Muscle fatty acid profiles (% of total fatty acids) of juvenile *C. labrosus* fed the control (CT) and *C. fusca* + *V. proteolyticus* (C + V) diets for 90 days.

Fatty Acids	CT	C + V	*p*
14:0	2.56 ± 0.04	2.41 ± 0.03 *	0.016
16:0	22.07 ± 0.36	22.29 ± 0.41	0.702
16:1n7	6.87 ± 0.13	5.99 ± 0.69	0.278
18:0	3.94 ± 0.10	3.74 ± 0.04	0.085
18:1n7	1.20 ± 0.07	1.15 ± 0.03	0.572
18:1n9	30.33 ± 0.58	28.03 ± 0.33 *	0.008
18:2n6	11.35 ± 0.48	10.77 ± 0.12	0.303
18:3n3	1.90 ± 0.12	2.11 ± 0.09	0.195
20:1n9	1.41 ± 0.23	1.19 ± 0.08	0.418
20:4n6, ARA	0.37 ± 0.08	0.56 ± 0.06 *	0.024
20:5n3, EPA	2.74 ± 0.26	3.42 ± 0.14 *	0.048
22:5n3	2.14 ± 0.46	1.38 ± 0.07	0.175
22:6n3, DHA	4.67 ± 0.27	6.95 ± 0.64 *	0.011
SFA	28.58 ± 0.48	28.44 ± 0.45	0.841
MUFA	39.80 ± 0.49	36.37 ± 0.94 *	0.012
PUFA	24.98 ± 0.87	27.09 ± 0.87	0.125
Other FA	4.81 ± 0.31	5.90 ± 1.02	0.357
n-3	13.26 ± 0.35	15.76 ± 0.86 *	0.027
n-6	11.72 ± 0.53	11.33 ± 0.09	0.510
n-9	31.73 ± 0.39	29.22 ± 0.34 *	0.001
n- 3 PUFA	10.58 ± 0.19	13.07 ± 0.83 *	0.042
n-3/n-6	1.14 ± 0.03	1.39 ± 0.08 *	0.014
EPA/DHA	0.58 ± 0.02	0.51 ± 0.04	0.137
PI ^1^	88.65 ± 2.41	114.84 ± 2.40 *	<0.001
IT ^2^	0.43 ± 0.01	0.39 ± 0.01 *	0.010
IA ^3^	0.50 ± 0.01	0.50 ± 0.01	0.612
FLQ ^4^	6.84 ± 0.27	9.31 ± 0.28 *	0.023

Dietary codes: CT, control diet; C + V, *C. fusca* + *V. proteolyticus* supplemented diet. Values are expressed as mean ± SEM (standard error of the mean) of 9 fish per treatment. Asterisks denote significant differences (*p* < 0.05). SFA: saturated fatty acids; MUFA: monounsaturated fatty acids; PUFA: polyunsaturated fatty acids; ARA: arachidonic acid; EPA: eicosapentaenoic acid; DHA: docosahexaenoic acid; ^1^ PI: peroxidability index = (% monoenoic × 0.025) + (% dienoic × 1) + (% trienoic × 2) + (% tetraenoic × 4) + (% pentaenoic × 6) + (% hexaenoic × 8); ^2^ IT: index of thrombogenicity = (14:0 + 16:0 + 18:0)/[(0.5 × 18:1) + (0.5 × ΣMUFAs) + (0.5 × n-6 PUFAs) + (3 × n-3 PUFAs) + (n-3/n-6)]; ^3^ IA: index of atherogenicity = (12:0 + 4 × 14:0 + 16:0)/[(n-6 + n-3) PUFAs + 18:1 + other MUFAs]; ^4^ FLQ (%): fish lipid quality = [(20:5n3 + 22:6n3)/total lipid] × 100.

**Table 6:** Plasma and liver metabolites in juvenile *C. labrosus* fed the control (CT) and *C. fusca* + *V. proteolyticus* (C + V) diets for 90 days.

Parameters	CT	C + V	*p*
*Plasma*			
Glucose (mM)	7.19 ± 0.36	8.90 ± 0.26 *	0.018
Triglycerides (mM)	0.27 ± 0.01	0.34 ± 0.01 *	0.004
Lactate (mM)	1.97 ± 0.15	1.77 ± 0.06	0.263
*Liver*			
Glucose (mmol g^−1^w.w.)	14.97 ± 0.65	14.74 ± 0.24	0.730
Glycogen (mmol g^−1^w.w.)	131.13 ± 3.91	110.59 ± 1.36 *	0.002
Triglycerides (mmol g^−1^w.w.)	52.66 ± 3.79	69.78 ± 2.08 *	0.018
Lactate (mmol g^−1^w.w.)	0.92 ± 0.05	1.62 ± 0.10 *	0.001

Dietary codes: CT, control diet; C + V, *C. fusca* + *V. proteolyticus* supplemented diet. Values are expressed as mean ± SEM (standard error of the mean) of 9 fish per treatment. Asterisks denote significant differences (*p* < 0.05). Values are expressed as grams of the wet tissue weight (g^−1^ w.w.).

**Table 7:** White and red muscle metabolites in juvenile *C. labrosus* fed the control (CT) and *C. fusca* + *V. proteolyticus* (C + V) diets for 90 days.

Parameters	White Muscle		Red Muscle		Two-Way ANOVA		
	CT	C + V	CT	C + V	Diet	Type of Muscle	Diet × Type of Muscle
Glucose (mmol g^−1^w.w.)	8.27 ± 0.35 ^a^	7.02 ± 0.31 ^a^	11.46 ± 0.66 ^b^	11.12 ± 0.23 ^b^	0.072	˂0.0001	0.289
Glycogen (mmol g^−1^w.w.)	8.20 ± 1.19 ^a^	16.62 ± 1.23 ^a^	42.43 ± 4.26 ^c^	32.18 ± 2.22 ^b^	0.740	˂0.0001	0.002
Triglycerides (mmol g^−1^w.w.)	4.53 ± 0.73 ^a^	4.66 ± 0.41 ^a^	51.01 ± 2.56 ^b^	52.88 ± 0.83 ^b^	0.485	˂0.0001	0.544
Lactate (mmol g^−1^w.w.)	40.83 ± 0.47 ^a^	39.26 ± 0.39 ^a^	16.62 ± 0.83 ^c^	22.38 ± 1.01 ^b^	0.010	˂0.0001	˂0.001

Dietary codes: CT, control diet; C + V, *C. fusca* + *V. proteolyticus* supplemented diet. Values are expressed as mean ± SEM (standard error of the mean) of 9 fish per treatment. *p*-values from two-way ANOVA (*p* < 0.05). The Tukey post-hoc test was used to identify differences in the experimental treatments. Different letters in a row denote significant differences for a given type of muscle between groups fed different diets. Values are expressed as grams of the wet tissue weight (g^−1^ w.w.).

**Table 8:** Digestive enzyme activity (U g tissue^−1^) measured in the intestinal extracts obtained from the proximal and distal intestinal regions of juvenile *C. labrosus* fed the control (CT) or the *C. fusca* + *V. proteolyticus* (C + V) diets for 90 days.

	CT	C + V	*p*
*Proximal intestine*			
Total alkaline protease	1260.01 ± 55.29	1989.01 ± 73.67 *	<0.001
Trypsin	0.69 ± 0.03	0.74 ± 0.03	0.286
Chymotrypsin	10.06 ± 0.43	11.57 ± 0.47	0.747
Leucine aminopeptidase	0.61 ± 0.02	0.75 ± 0.02 *	<0.001
Alkaline phosphatase	3.23 ± 0.15	5.64 ± 0.21 *	<0.001
*Distal intestine*			
Total alkaline protease	1174.07 ± 43.61	1921.06 ± 51.14 *	<0.001
Trypsin	0.57 ± 0.03	0.75 ± 0.02 *	0.001
Chymotrypsin	10.26 ± 0.51	10.73 ± 0.42	0.505
Leucine aminopeptidase	0.65 ± 0.02	0.90 ± 0.02 *	<0.001
Alkaline phosphatase	3.10 ± 0.13	5.48 ± 0.16 *	<0.001

Dietary codes: CT, control diet; C + V, *C. fusca* + *V. proteolyticus* supplemented diet. Values are expressed as mean ± SEM (standard error of the mean) of 12 fish per treatment. Asterisks denote significant differences (*p* < 0.05).

**Table 9:** Quantification of the histological parameters assessed (µm) in the proximal and distal intestinal regions of juvenile *C. labrosus* fed the control (CT) or *C. fusca* + *V. proteolyticus* (C + V) diets for 90 days.

	CT	C + V	*p*
*Proximal intestine*			
Fold length (µm)	656.65 ± 8.17	915.58 ± 8.95 *	<0.001
Fold diameter (µm)	138.12 ± 2.14	125.06 ± 2.13 *	<0.001
Serosa layer (µm)	24.78 ± 0.58	29.82 ± 1.04	<0.001
Mucosa layer (µm)	34.45 ± 1.11	33.58 ± 0.70	0.541
Submucosa layer (µm)	22.09 ± 0.70	18.94 ± 0.31 *	<0.001
Lamina propria (µm)	23.97 ± 0.63	14.87 ± 0.46 *	<0.001
*Distal intestine*			
Fold length (µm)	755.10 ± 14.07	653.97 ± 9.37	0.0558
Fold diameter (µm)	189.05 ± 3.85	116.31 ± 1.17 *	<0.001
Serosa layer (µm)	45.21 ± 1.22	27.01 ± 1.17 *	<0.001
Mucosa layer (µm)	31.39 ± 1.05	31.82 ± 1.09	0.783
Submucosa layer (µm)	38.47 ± 0.96	20.19 ± 0.59 *	<0.001
Lamina propria (µm)	49.72 ± 2.42	24.80 ± 0.97 *	<0.001

Dietary codes: CT, control diet; C + V, *C. fusca* + *V. proteolyticus* supplemented diet. Values are expressed as mean ± SEM (*n* = 50 measurements per treatment). Asterisks denote significant differences (*p* < 0.05) between experimental diets in the same intestinal region.

## Data Availability

The data presented in this study are available on request from the corresponding author.

## Supplementary Materials

The following supporting information can be downloaded at: https://www.mdpi.com/article/10.3390/ani13040589/s1, Figure S1: Comparison of EPA + DHA content (mg/100 g of raw meat) in wild specimens (García-Márquez et al. [56]), specimens fed 15% *C. fusca* (C-15) diet and their control group (CT EXP1) (García-Márquez et al. [37]), and specimens fed the combination of *C. fusca* and *V. proteolyticus* (C + V) and their control diet (CT EXP2) (the present study); Table S1: Quantification of the histological parameters assessed in the liver of juvenile *C. labrosus* fed the control (CT) or *C. fusca + V. proteolyticus* (C + V) diets for 90 days; Table S2: Increase in growth performance and nutrient utilization ratios in *C*. *labrosus* fed 90 days with 15% *C. fusca* (C-15) diet against its control diet (CT EXP1) (García-Márquez et al. [37]), and specimens fed 90 days the combination of *C. fusca* and *V. proteolyticus* (C + V) against its control diet (CT EXP2) (present study). Values represent the mean of each replicate tank against the mean of the controls (C-15/CT EXP1 and C + V/CT EXP2 quotients) in each parameter.
