# Supplementary material for: Evaluation of the Combined Administration of Chlorella fusca and Vibrio proteolyticus in Diets for Chelon labrosus: Effects on Growth, Metabolism, and Digestive Functionality
Source: Animals (Basel). 2023 Feb 7;13(4):589. doi: 10.3390/ani13040589 (PMC9951767; doi:10.3390/ani13040589)
Supplement: Supplementary file 1 [file animals-13-00589-s001.zip › Figure S1.pdf]

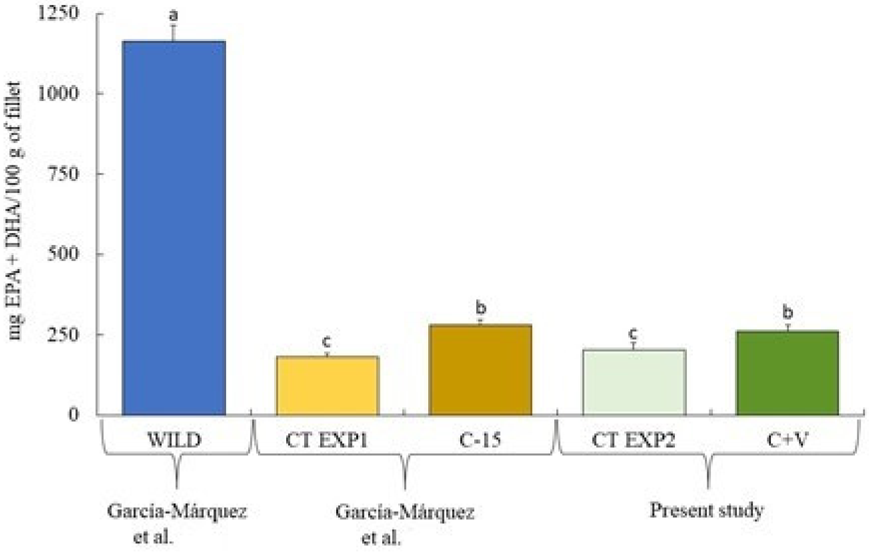

Figure S1. Comparison of EPA+DHA content (mg/100 g of raw meat) in wild specimens (García-Márquez et al. [56]), specimens fed 15% *C. fusca* (C-15) diet and their control group (CT EXP1) (García-Márquez et al. [37]), and specimens fed the combination of *C. fusca* and *V. proteolyticus* (C+V) and their control diet (CT EXP2) (the present study).
