# Supplementary material for: Evaluation of the Combined Administration of Chlorella fusca and Vibrio proteolyticus in Diets for Chelon labrosus: Effects on Growth, Metabolism, and Digestive Functionality
Source: Animals (Basel). 2023 Feb 7;13(4):589. doi: 10.3390/ani13040589 (PMC9951767; doi:10.3390/ani13040589)
Supplement: Supplementary file 1 [file animals-13-00589-s001.zip › Table S1.pdf]

Table S1. Quantification of the histological parameters assessed in the liver of juvenile *C. labrosus* fed the control (CT) or *C. fusca* + *V. proteolyticus* (C+V) diets for 90 days

	**CT**	**C+V**	***p***
Hepatocyte area (μm^2)	13.19 ± 0.25	12.62 ± 0.22	0.108
Hepatocyte major axis (μm)	4.78 ± 0.06	4.92 ± 0.08	0.225

Dietary codes: CT, control diet; C+V, *C. fusca* + *V. proteolyticus* supplemented diet. Values are expressed as mean ± SEM (n = 50 measurements per treatment).
