# Supplementary material for: Evaluation of the Combined Administration of Chlorella fusca and Vibrio proteolyticus in Diets for Chelon labrosus: Effects on Growth, Metabolism, and Digestive Functionality
Source: Animals (Basel). 2023 Feb 7;13(4):589. doi: 10.3390/ani13040589 (PMC9951767; doi:10.3390/ani13040589)
Supplement: Supplementary file 1 [file animals-13-00589-s001.zip › Table S2.pdf]

Table S2. Increase in growth performance and nutrient utilization ratios in *C. labrosus* fed 90 days with 15 % *C. fusca* (C-15) diet against its control diet (CT EXP1) (García-Márquez et al. [37]), and specimens fed 90 days the combination of *C. fusca* and *V. proteolyticus* (C+V) against its control diet (CT EXP2) (present study). Values represent the mean of each replicate tank against the mean of the controls (C-15 /CT EXP1 and C+V/CT EXP2 quotients) in each parameter.

Parameters	C-15/CT EXP1	C+V/CT EXP2	*p*
**WG**	1.07 ± 0.01	1.04 ± 0.06	0.623
**SGR**	1.10 ± 0.03	1.14 ± 0.05	0.424
**FCR**	0.76 ± 0.02	0.79 ± 0.10	0.837
**PER**	1.32 ± 0.05	1.46 ± 0.09	0.194

Dietary codes: C-15 and CT EXP1, fish fed with 15 % *C. fusca* and control diet, respectively (both from García-Márquez et al. [37]); C+V and CT EXP2, fish fed with *C. fusca* and *V. proteolyticus* and control diet, respectively (both from the present work). Data are expressed as mean ± SEM (standard error of the mean) of triplicate tanks. WG: weight gain (%) = (final fish weight – initial fish weight) x 100; SGR: specific growth ratio (% day^{-1}) = 100 x [(ln final fish weight) - (ln initial fish weight)]/experimental days; FCR: feed conversion ratio = dry feed intake (g)/weight gain (g); PER: protein efficiency ratio = weight gain/intake of dietary protein.
